# On the Evidence Supporting That AN11127 Encodes an Aspergillus Nidulans Sec12 Orthologous Protein. Reply to Bravo-Plaza et al. Comment on “Dimou et al. Profile of Membrane Cargo Trafficking Proteins and Transporters Expressed under N Source Derepressing Conditions in *Aspergillus nidulans*. *J. Fungi* 2021, *7*, 560”

**DOI:** 10.3390/jof7121040

**Published:** 2021-12-03

**Authors:** Sofia Dimou, Xenia Georgiou, Eleana Sarantidi, George Diallinas, Athanasios K. Anagnostopoulos

**Affiliations:** 1 Department of Biology, National and Kapodistrian University of Athens, Panepistimioupolis, 15784 Athens, Greece; sodimou@biol.uoa.gr (S.D.); geoxenia99@gmail.com (X.G.); 2 Proteomics Research Unit, Biomedical Research Foundation of the Academy of Athens (BRFAA), 11527 Athens, Greece; eleanasarantidi@gmail.com; 3 Institute of Molecular Biology and Biotechnology, Foundation for Research and Technology, 70013 Heraklion, Greece

Prof. Peñalva and co-workers provided evidence that AN11127 is related by sequence and function to Sec12 [1,2]. In our recent article [3], we expressed the opinion, that AN11127 might not be an orthologue of functionally characterized Sec12 proteins in yeast or mammals. Our opinion was based on the lack of detection of significant protein similarity using *blastp* analysis of the AN11127 protein in genome databases and also on the observations that several Aspergilli, and ascomycetes in general, lack AN11127-similar proteins. On this basis, we expressed the hypothesis that AN11127 might be a functional analogue of other Sec12 proteins. In no point in our article did we doubt that the product of AN11127 functions as Sec12-like guanine nucleotide exchange factor (GEF) specific for SAR1 in *A. nidulans* [1]. 

Prof. Peñalva and co-workers provided several lines of evidence, including novel in silico searches, supporting that AN11127 is the Sec12 protein of *A. nidulans.* The authors in Section 2.1 provide evidence showing that AN11127 is isofunctional to other Sec12 proteins. Yet, functional analogy is not evidence for orthology [4]. Thus, the entire Section 2.1 provides no evidence determining whether Sec12 is functionally analogous or orthologous to known Sec12 proteins in other organisms. A similar biochemical function, similar subcellular topology, a shared common motif, the presence of a transmembrane segment, and essentiality for viability, are all expected aspects in proteins that are isofunctional in COPII formation and function [5], and not necessarily orthologous.

In the second part of the Results Section (Section 2.2), Prof. Peñalva and co-workers used NCBI tblastn in FungiDB to search for genomic sequences encoding AN11127 product homologues. Their new searches provided convincing evidence that AN11127 is present in possibly all Aspergilli and most ascomycetes. Their targeted search indeed “corrected” our inability to detect AN11127 homologues in several fungi using the standard blastp search and also highlighted the dangers of false annotation of genome databases. We welcome this new evidence that orthologues of AN11127 seem to exist in most ascomycetes. 

On the positive side, the discussion raised by our article highlights the evolutionary particularity of Sec12-like proteins in fungi, plants and Metazoa, as they are exceptionally divergent in sequence in different species or genera, seemingly more divergent than any other of the main proteins involved in COPII formation and function (e.g., Sar1, Sec24, Sec23, Sec13 and Sec31 [5]). The high divergence of Sec12-like proteins, which might also hide cryptic convergent evolution events, is interesting from both the evolutionary and functional points of view.
